# Honing the Double-Edged Sword: Improving Human iPSC-Microglia Models

**DOI:** 10.3389/fimmu.2020.614972

**Published:** 2020-12-08

**Authors:** Anne Hedegaard, Szymon Stodolak, William S. James, Sally A. Cowley

**Affiliations:** Sir William Dunn School of Pathology, University of Oxford, Oxford, United Kingdom

**Keywords:** microglia, human, induced pluripotent stem cells, *in vitro* models, media composition, 3D scaffolds, co-culture, physiological relevance

## Abstract

Human induced Pluripotent Stem Cell (hiPSC) models are a valuable new tool for research into neurodegenerative diseases. Neuroinflammation is now recognized as a key process in neurodegenerative disease and aging, and microglia are central players in this. A plethora of hiPSC-derived microglial models have been published recently to explore neuroinflammation, ranging from monoculture through to xenotransplantation. However, combining physiological relevance, reproducibility, and scalability into one model is still a challenge. We examine key features of the *in vitro* microglial environment, especially media composition, extracellular matrix, and co-culture, to identify areas for improvement in current hiPSC-microglia models.

## Introduction

Microglia represent a branch of tissue-resident macrophages which originate primarily in the yolk sac (1) and finally mature in the central nervous system (CNS) (2, 3). Once within the CNS, microglia function as important homeostatic cells and innate immune sentinels, attracting the analogy of a double-edged sword through their contribution to both health and disease. They influence developing neural networks by regulating the rate of neurogenesis and pruning neuronal synapses [(4), reviewed by (5)]. As ramified cells, they aid brain homeostasis by surveying the brain parenchyma and clearing cellular debris. However, as amoeboid cells, they secrete cytokines in response to pathogens (e.g. viral infection), misfolded proteins (e.g. Alzheimer’s disease), or cellular damage (e.g. brain injury). The cytokine response can exacerbate neuronal damage if prolonged, contributing to chronic neurodegenerative disease [(6), reviewed by (7)]. This key pathological process is still inadequately understood, hindered by the lack of good quality *in vitro* models.

## Why Use hiPSC-Microglia to Model Microglia?

Human induced Pluripotent Stem Cells (hiPSC) offer unprecedented advantages over other commonly used primary and immortalized cell cultures, notably human genetic background, normal karyotype, limitless self-renewal, and suitability for gene editing [reviewed by (8)]. The human genetic background solves the poor representation of disease-relevant gene orthologs in mouse models (9, 10) deemed partly responsible for the poor success-rate of translating therapeutic modalities from mouse studies into the clinic [reviewed by (11, 12)]. Many neurological diseases are multigenic, and therefore particularly difficult to model in mice, whereas hiPSC enable representation of extremes of polygenic risk score (13), and endogenous levels of protein expression.

Microglial identity is driven by both developmental origin and brain environment [reviewed by (14)]. They derive from early waves of primitive yolk sac macrophages, which migrate to the brain rudiment even before neural progenitors develop (1, 15), and are maintained by local self-renewal throughout life (16). Several strategies have been published recently to derive microglia from hiPSC, which generally mirror the main characteristics of primitive hematopoiesis, including dependence on PU.1 and IRF8 transcription factors and MYB independence [(15, 17) reviewed by (18)], though the *in vitro* developmental pathway does not necessarily conform exactly to *in vivo* primitive waves. While mesoderm/hemogenic endothelium/myeloid differentiation can be achieved using simple growth factor cocktails (minimally BMP4, VEGF, SCF, followed by IL-3, M-CSF), mature microglial identity subsequently relies on cues present in the CNS environment, and this is where protocols differ in terms of their final medium composition and use of monoculture *versus* co-culture to induce microglial maturation (19–28). Some protocols have been more widely adopted and implemented within independent labs and used at scale by companies, making them highly reproducible [see (19, 26, 29) and (25) adopted by (27, 30)].

Within hours of isolation from the brain, both human and rodent primary microglia downregulate several key mature microglial markers, particularly TMEM119, P2RY12, and SALL1 (2, 31), which can be restored upon re-transplantation into the brains of microglia-deficient mice (32). Likewise, hiPSC-microglia assume the closest identity to *in vivo* microglia when they are transplanted into rodent brains (9, 19, 33–35). However, this is clearly not practical for high-throughput experiments, and is still limited by the xenogenic environment. Therefore, further improving *in vitro* hiPSC-microglia models to better represent their *in vivo* counterparts remains paramount. The pros and cons of different hiPSC-microglia models, and their application for disease modeling, have been reviewed recently (18, 36–39), but how to improve the physiological relevance of these models is an under-covered area. In this mini review, we focus on how to better mimic the CNS habitat, with relevant media composition, extracellular matrix, and co-culture, which will further improve the physiological applications and reproducibility of existing *in vitro* hiPSC-microglia protocols.

## Improving hiPSC-Microglia Media Composition

To better understand, compare, and control hiPSC-microglia, we need fully defined, open-source media, which not only supply relevant survival/differentiation factors, but also reflect the composition of the brain interstitial fluid, mimicking ionic composition, energy-substrates, nutrients, minerals, vitamins, pH, and osmolarity. However, many conventional media compositions are not at all physiological, but rather a hangover from culturing cancer cell lines with high metabolic and proliferative rates. Firstly, media with high levels of glucose (up to 25 mM, *versus* physiological levels of 5 mM), can mask cellular phenotypes (40, 41), which is problematic, as microglial metabolic dysregulation is associated with disease phenotype (42). Secondly, while serum is a major source of nutrients, and its use made cell-culture of microglia possible in the first place (43), it is still often used for *in vitro* culture of glial cells, despite the fact that glia are not exposed to serum under normal conditions *in vivo*, as they are separated by the blood-brain barrier [reviewed by (44)]. Further, each serum batch is different, its composition is undefined, and it contains lipopolysaccharide (LPS)-binding proteins which aid binding to toll-like receptor 4 (TLR4), thereby enhancing microglial responses to LPS (45). Thirdly, a hangover from explant cultures are components which contain immunosuppressive molecules, notably B27 supplement, developed to prevent excessive cell death and reactivity, but which interfere with microglia responses (46).

Defined, more physiological media now exist [e.g. BrainPhys (47)], and serum-free conditions have been demonstrated to be viable for rodent microglia with the recently developed “TIC media.” TIC supplements DMEM/F12 with astrocyte-derived factors TGF-β, IL-34, and cholesterol (32). TGF-β promotes maturation and specialization of microglia within the developing brain, helps maintain the identity of cultured microglia [both when isolated as primary cells (2, 31) or derived from human iPSCs (19)], and encourages an anti-inflammatory quiescent microglia phenotype (48). IL-34 is the main ligand in the brain for Colony Stimulating Factor 1 receptor (CSF-1R), signaling through which is essential for microglial survival (1, 49). Cholesterol further improved the benefit conveyed by IL-34/TGF-β, despite myeloid cells being capable of *de novo* cholesterol synthesis [(50) reviewed by (51)]. However, despite TIC factors enabling survival, serum was still required to initiate microglial phagocytic activity (32). Overall, whilst the field is clearly moving towards defined, physiological media for hiPSC-microglia, serum confounds this, and use of immunomodulatory additives needs to be noted when comparing inflammatory responses across studies.

## Improving hiPSC-Microglia Through Scaffolded 3D Extracellular Environment

Microglia *in vivo* are embedded in a three-dimensional network of macromolecules derived from both neuronal and glial cells, mainly glycosaminoglycans (e.g. hyaluronic acid), proteoglycans, glycoproteins, and low levels of fibrous proteins (including collagen, laminin, fibronectin, and vitronectin), creating a rather “soft,” viscoelastic brain environment of ~3 kPa (52, 53). Unfortunately, these basic features are mostly absent in conventional *in vitro* conditions. Mimicking this 3D extracellular environment should enable hiPSC-microglia to adopt a more authentic phenotype. This is particularly important as immune cells are especially prone to develop unwanted and inflammatory responses to a suboptimal environment, such as covalently cross-linked (purely elastic) synthetic matrices, which lack viscoelastic properties [(54), reviewed by (55)].

3D cell culture, both scaffolded and scaffold-free, is becoming more widely used, as it preserves natural cell shape, supports cell-to-cell and cell-to-matrix communication, enhances cell differentiation, and pushes gene and protein expression towards that found *in vivo* [reviewed by (56)]. Surprisingly, only a few groups have cultured microglial cells in a 3D scaffolded environment (**Table 1**), and several of them primarily focus on other cell types co-cultured with microglia, including neurons (70) and glioblastoma cells (71, 72). To properly distinguish the effects attributable to 3D conditions, systematic studies using relevant 2D controls are required for appropriate comparisons to be made.

**Table 1:** Key studies of microglial culture in 3D scaffolds.

	Material	Modifications	Serum	Microglia	Observations	Reference
**NATURAL HYDROGEL AND DERIVATIVES**	Alginate	RGD sequence	√	Fetal rat primary	1. Amoeboid morphologies over 2 weeks	Frampton et al. (57)
	Methacrylated hyaluronic acid	×	√	Postnatal rat primary	1. Heterogenous morphology (mostly amoeboid) 2. Microglia wrapped processes around other cells—perhaps phagocytosis or cell−cell interaction	Jeffery et al. (58)
	Hyaluronic acid	Combined with basal lamina mixture	√	Postnatal rat primary	1. Microglia in 3D hyaluronic acid hydrogel mostly small with few processes 2. Microglia in 3D hyaluronic acid lamina hydrogel more dispersed and more thin processes	Koss et al. (59)
	Hyaluronic acid-gelatin	Combined with Gelin-S and heparin	√	Human fetal immortalized CHME3	1. Protective effect of microglia on GBM greater in the 3D model when challenged with cytotoxics 2. Microglia promoted GBM proliferation more in 3D *vs* 2D	Leite et al. (60)
	Collagen I	×	√	Murine immortalized BV-2	1. 3D collagen promotes multiplanar projections 2. Increased inflammatory response following LPS stimulation *versus* 2D collagen coating	Haw et al. (61)
	Collagen I	×	√	Murine immortalized BV-2	3D collagen hydrogel *vs* 2D uncoated: 1. Smaller fold change in gene expression following LPS treatment 2. Smaller fold changes in ROS production following LPS treatment	Cho et al. (62)
	Matrigel	×	√	Human immortalized SV40	1. Microglia seeded in 3D Matrigel in an angular chamber presented protrusions 2. Microglia only migrated to the central chamber with neurons and astrocytes when neural cells were overexpressing Aβ 3. Once together with neurons and astrocytes, microglia were actively in contact with neurons and astrocytes by expanding and retracting their protrusions	Park et al. (63)
**SYNTHETIC HYDROGEL & OTHERS**	Synthetic peptide	Fibronectin or collagen I coating	√	Murine immortalized BV-2	1. Peptide hydrogel promoted ramification 2. Fibronectin or collagen addition promoted deramification	Pöttler et al. (64)
	Graphene foam	×	√	Murine immortalized BV-2	3D graphene *vs* 2D graphene: 1. did not induce further microglial ramification 2. decreased NO production following LPS stimulation 3. Conditioned medium (CM) promoted greater survival of mouse primary neural stem cells 5. CM rescued LPS-induced neuroinflammation	Song et al. (65)
	PEG	MMP-degradable peptide cross-links and CRGDS sequence peptide	×	Derived from human ESC line H1	1. Microglia adopted both ramified and ameboid morphologies in 3D co-culture with other brain cell types	Schwartz et al. (66)
	Graphene foam	×	√	Murine immortalized BV-2	1. CM from microglia in 3D graphene (*vs* 2D graphene) promoted neurosphere formation, facilitated mouse primary NSC migration from neurospheres, and increased single cell polarization by activating the SDF-1α/CXCR4 signaling pathway and enhanced cell adhesion on the substrate.	Jiang et al. (67)
	Fmoc–Phe3 peptide	×	√	Postnatal rat primary	1. Fmoc–Phe3 peptide hydrogel stimulated microglial proliferation and NGF secretion (due to the peptide, not 3D conditions)	Chronopoulou et al. (68)
	P(TMC-CL)	×	√	Postnatal rat primary	1. Cells cultured within a 3D P(TMC-CL) scaffold were smaller and with elongated processes compared to cells cultured on a P(TMC-CL) flat film. 2. Following exposure to myelin, only cells in 2D conditions presented abberant multinucleated phenotype	Pires et al. (69)

Aβ, amyloid beta; CM, conditioned media; CRGDS, peptide sequence of Cys-Arg-Gly-Asp-Ser present in fibronection; CXCR4, C-X-C chemokine receptor 4; ESC, embryonic stem cell; GBM, glioblastoma multiforme; LPS, lipopolysaccharide; MMP, matrix metalloproteinase; NGF, nerve growth factor; NO, nitric oxide; NSC, neural stem cell; PEG, polyethylene glycol; P(TMC-CL), poly(trimethylene carbonate-co-ϵ-caprolactone); RGD, tripeptide motif of Arg-Gly-Asp present in fibronectin; SDF-1α, stromal cell-derived factor 1 α.

Scaffolded 3D culture influences microglial morphology and attachment, but the outcome depends on the scaffold composition and structure. Choosing a material that optimizes microglial phenotype still remains a challenge. Hydrogels of synthetic peptide (68), collagen (61, 64), or PEG (66) enhanced ramification in both postnatal rat microglia and immortalized murine BV-2 cells. Likewise, primary rat microglia cultured within a 3D fibrous poly(trimethylene carbonate-co-ϵ-caprolactone) scaffold had smaller cells with elongated processes *versus* cells attached to flat solvent-cast films (69). However, this was not observed in 3D graphene scaffolds, where BV-2 cells remained mostly amoeboid (65). Some peptides, like neural cell adhesion molecule (NCAM)-derived KHIFSDDSSE, favor neuron and astrocyte attachment but not microglial attachment (73). Functionalizing hydrogels with extracellular matrix (ECM) components have provided mixed results; a basal lamina mixture prompted primary postnatal rat microglia within a glial co-culture to disperse throughout the hyaluronic acid-based scaffold (59), whereas coating a collagen hydrogel with fibronectin and laminin decreased ramification in BV-2 cells (64).

3D culture can also affect microglial behavior and interaction with other cell types. Neuronal survival in conditioned media from 3D-cultured BV-2 cells was higher than from 2D-cultured cells, and LPS-induced BV-2-mediated neuronal toxicity was also reduced as a result of 3D microglial culture (65). Meanwhile, more BV-2 cells upregulated CD40 (induced through NF-κB and STAT-1α) in response to LPS when in 3D collagen than in 2D (61), suggesting a more homogenous inflammatory response in 3D (74). Although BV-2 and other immortalized cell lines have been a valuable resource for studying microglia, it is likely that microglial responses to a 3D environment might be masked or even reversed by the aberrantly proliferative and primed state of such cells (75, 76). Nonetheless, 3D culture of microglia in specific scaffolds seems to promote a production of growth factors and an anti-inflammatory phenotype beneficial to other cell types.

## Improving hiPSC-Microglia Through Co-Culture

The simplest approach to study cross-talk between microglia and neurons or astrocytes, is by transferring medium conditioned by these cells cultured separately. This provides a high level of experimental control, as the conditioned media can be analyzed independently to identify cytokines produced by stimulated microglia (77), assess the impact of individual factors on survival (32), or interrogate the process studied using drugs. However, conditioned media only allows study of uni-directional signaling. Transwells or Boyden chambers allow different cell types to exchange secreted factors bi-directionally [reviewed by (78)]. This improves physiological relevance but offers less insight into which cell type the secreted signals originated from, or control over which cells are targeted with any manipulation or drug given. Furthermore, these culture methods are also limited by the effective concentration of secreted signals, being much lower compared to local concentrations if cells were in physical contact.

**Figure 1:**
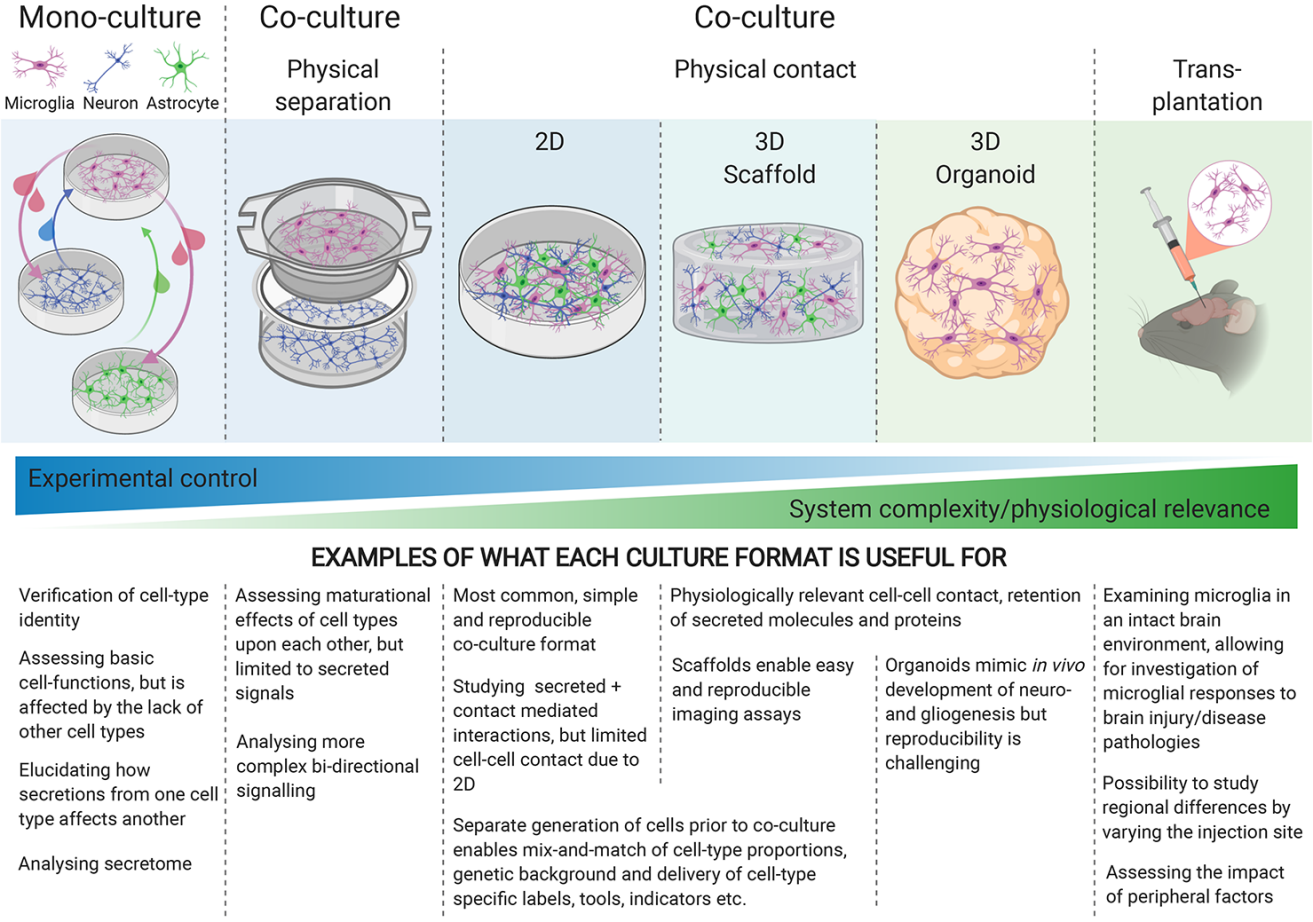
Different culture environments convey different levels of experimental control and physiological relevance, with mono-cultures at one end of the spectrum, and transplantation into intact brains at the other. The experimental question being investigated should guide the choice of culture-system.

To fully capture the physiology of cellular interactions, physical contact-mediated cues, through receptor-ligand interactions, are required. Microglial quiescence is maintained through neuronal CD200 glycoprotein interaction with the microglial CD200 receptor [(79) reviewed by (80)], as well as neuronal transmembrane (but possibly also secreted) CD22 interaction with microglial CD45 receptors (81). The mainly neuronal chemokine CX3CL1 (“fractalkine”) maintains microglia in a surveying state when the membrane-bound form makes contact with the microglial CX3CR1 receptors, whereas soluble CX3CL1 has a chemoattractant effect on microglia [(82), reviewed by (83)]. Furthermore, the idea that electrical activity from neurons might play a role in immune-response suppression has been around for two decades (84), but the nature of this interaction is not well understood. Generating physical-contact co-cultures would evidently enable microglia to experience all modes of interaction with other cell types, yet when increasing the physiological relevance of the culture model, the degree of control over the experimental system decreases, highlighting the importance of choosing the appropriate culture-model for each research question (**Figure 1**).

Most hiPSC-microglia studies have focused on characterizing the gene expression and functionality of the resulting microglia in monoculture [reviewed by (8, 36)], but a few have included co-cultures with neurons (19, 24, 27) in 2D (3D co-cultures will be discussed later). Physical contact with neurons increases the expression of mature microglial genes, enhances ramified morphology compared to conditioned media, as well as eliciting a dampened response to LPS + IFNγ, high microglial motility and extension of processes to sites of injury (19, 24, 27), reflecting the homeostatic surveillance and response to injury associated with microglia *in vivo* (85). The exact cues responsible for these phenotypes are yet to be elucidated. Our understanding of interactions between microglia and astrocytes is limited, these cells typically being examined in isolation, but interest in their complex relationship, in terms of maintaining homeostasis in health and reactive cross-talk in disease, is growing [reviewed by (86)]. Pandya et al. used astrocytes as a feeder layer for maturing their hiPSC-microglia for 1–2 weeks before isolating the CD39^+^ microglia for monoculture (23), but interactions between mature hiPSC-microglia and astrocytes are otherwise currently under-explored.

The maturity and functionality of the non-microglial co-cultured cells is important to consider, as crucial microglial functions like synaptic pruning are likely only observable if the synaptic network is sufficiently mature, which may depend on more than bi-culture. Tri-culture of microglia with neurons and astrocytes will enable further maturation, more fully capture additional aspects of cross-talk and identify potential compensatory mechanisms between different brain cell types. Microglia and astrocytes operate in concert, both possessing that “double-edged sword” capacity to either support neuronal recovery (simplistically, “helpful” M2 and A2 phenotypes) or cause neuronal death (“harmful” M1 and A1 reactive phenotypes) [(77) reviewed by [87)]. A primary rodent tri-culture system of neurons, astrocytes and microglia in serum-free conditions (using TIC-supplemented media) shows promise in modeling different neuroinflammatory scenarios (88), in line with both *in vivo* and *in vitro* studies (77, 89, 90). However, TIC factors could not be omitted in the tri-cultures (88), indicating that endogenous secretion from the cultured astrocytes was not sufficient to retain a viable and physiologically active microglial phenotype. Interestingly, co-culture with hiPSC-neurons and astrocytes is capable of shifting the transcriptional profile of hiPSC-microglia further towards an *ex vivo* state than TIC media, although the gap between *in vitro* and *ex vivo* microglia is not fully closed (29).

Oligodendrocytes, endothelial cells, and/or vasculature are also likely to affect microglial function, yet they are rarely included in *in vitro* culture models, or assessment of their impact on microglia is lacking (66). Excitingly though, with the advent of single-cell RNAseq and single-cell proteomics, analyzing complex co-culture systems has become more straightforward and informative. These high-throughput methods allow for detection of overall cellular changes and activation/inactivation of biological pathways within each cell type, providing important clues to understand inter-cellular cross-talk. Using hiPSC, it is now possible to “mix and match” the genetic background of each cell type within the co-culture, such that the effect of a mutation can be studied in one cell type at a time. Strategies for co-culturing multiple hiPSC cell-types are increasingly popular and are beginning to move towards three dimensional environments as well.

## Combining Co-Culture and 3D Environment for hiSC-Microglia

Providing microglia with a more physiologically relevant cellular environment relies on the principle of letting separately derived microglia populate pre-existing 3D neuronal cultures, brain organoids, or xenotransplantation into rodent brains [reviewed by (18)]. 3D scaffolded cultures confer a high degree of reproducibility and experimental flexibility, as individual cell types can be derived separately and added to the scaffold one at a time. This enables mix and match of co-cultures, i.e. cell types, ratios, genetic backgrounds, genetic tools, and fluorescent reporters. A 3D scaffold culture with microfluidic chambers has demonstrated that immortalized human SV40-derived microglia display ramified morphologies when seeded within Matrigel, migrate towards Amyloid-beta (Aβ)-expressing neurons, and cleave their axons while producing neurotoxic mediators (63). This encouraging study could be made more physiologically relevant by replacing the genetically aberrant SV40-microglial cells with hiPSC-derived microglia, and mouse sarcoma-derived Matrigel with a defined hydrogel more reminiscent of brain ECM.

3D organoids preserve the human model context, yet high variability between organoid batches remains a technical challenge. Remarkably, cerebral organoids spontaneously containing microglia have been obtained (91), but this is only apparent when mesoderm is not sufficiently suppressed. More conventionally, organoids and microglia are derived separately, due to the differing ontogeny between ectodermally derived CNS cells and mesodermally derived microglia. Integrated microglia adopt ramified morphologies, display migration to the site of injury (19, 20, 28) and phagocytose Aβ (92).

Xenotransplantation offers the benefit of transplanting hiPSC-derived microglia into a more mature and structurally developed environment. However, immune-deficiency and expression of human CSF1 or IL34 are crucial for survival of the transplanted human microglia (33, 93), and ablation of host microglia is usually preferable. Excitingly, several recent xenotransplantation studies have detected improved hiPSC-microglial morphologies, with transcriptional profiles showing higher degrees of similarity between xenotransplanted microglia and freshly isolated human microglia than with cultured primary microglia (9, 33, 34). Meanwhile, the pattern of differentially expressed genes revealed a more muted response to LPS in transplanted cells than in *in vitro* cultured microglia (33). Engrafted microglia associate with Aβ plaques, appear to phagocytose them (19, 33), and upregulate disease-associated microglia markers, including CD9, MERTK, and TREM2 (33). Xenotransplantation is therefore a viable option for studying hiPSC-derived human microglia allowed to mature in an *in vivo* brain environment, where the issue of altered *in vitro* gene expression (31, 32) appears to have been resolved, but making this technique amenable to large scale-up will be extremely challenging.

## Discussion

Strides towards more physiological hiPSC-microglia culture conditions are being made apace, including the use of defined serum-free media in monoculture, the implementation of co-cultures, whether in 2D format, organoids, or xenotransplantation. One important underdeveloped gap in the repertoire is an *in vitro* model containing multiple hiPSC types embedded in a defined 3D scaffold, which would both enhance microglial identity and be amenable to advanced approaches, including arrayed drug screening, genome-wide CRISPR screens, high content imaging, cell multiplexing, and single cell transcriptomics. Further work is necessary to identify viscoelastic scaffold materials and ECM components that optimize microglial morphology, adhesion, and function. The process of neuroinflammation is arguably best modeled with all participating cell types present. Loss of function in astrocytes and/or microglia, impacting homeostatic support, or a toxic gain of function in the same cells, leading to the release of neurotoxic factors, would both probably have detrimental effects on neuronal health. Both scenarios are likely occurring in tandem in various neurodegenerative diseases, as well as in normal ageing, where defective phagocytosis as well as pro-inflammatory profiles have recently been reported (94, 95). Many links in the harmful inflammatory cascades that have been demonstrated to originate with microglia, propagate to astrocytes, and ultimately cause neurodegeneration, are not fully elucidated [(77), reviewed by (87)], and there is much more to learn about cross-talk and compensatory mechanisms between these cell types. Ultimately, the trade-off between the physiological relevance of a culture-system and experimental control over conditions must be decided based on the scientific question asked. Various hiPSC-microglia models will continue to be relevant, but the more defined and comparable we can make them, the better we will be able to understand and control the double-edged sword that is microglial function.

## Author Contributions

Conceptualization: SS, AH, and SC. Writing—Original Draft: SS and AH. Writing—Review and Editing: SS, AH, WJ, and SC. Funding Acquisition: WJ and SC. Supervision: WJ and SC. All authors contributed to the article and approved the submitted version.

## Funding

We thank the following for financial support: The Oxford Martin School for core support to the James Martin Stem Cell Facility (LC0910-004; SC); BBSRC industrial CASE studentship (BB/M011224/1; SS); Eli Lilly and Company (AH, SS). The authors declare that this study received funding from Eli Lilly and Company. The funder was not involved in the study design, collection, analysis, interpretation of data, the writing of this article or the decision to submit it for publication.

## Conflict of Interest

The authors declare that the research was conducted in the absence of any commercial or financial relationships that could be construed as a potential conflict of interest.
